# Endosymbiotic *Regiella insecticola* reduce mummification in native and transinfected aphid hosts but do not prevent reproductive damage post parasitism

**DOI:** 10.1098/rsos.261936

**Published:** 2026-10-28

**Authors:** Alex Gill, Joshua Thia, Kelly Richardson, Qiong Yang, Ary Hoffmann

**Affiliations:** School of BioSciences, Bio21 Molecular Science and Biotechnology Institute, The University of Melbourne, Melbourne, Australia

**Keywords:** *Diaereitella rapae*, *Aphidius colemani*, parasitism, fecundity

## Abstract

Some bacterial endosymbionts of aphids can reduce mummification rates following attack by parasitoids, yet these endosymbionts can remain at a low frequency in populations even when parasitoids are present. The endosymbiont *Regiella insecticola* is uncommon in the major pest aphid *Myzus persicae* despite providing apparent protection against parasitoid attack. Here, we consider population-level effects associated with *Regiella* in its native *M. persicae* host as well as *Regiella* transinfected into pest *Rhopalosiphum padi* (oat aphids) and *Diuraphis noxia* (Russian wheat aphids), focusing on two parasitoids (*Diaeretiella rapae*, *Aphidius colemani*). We consider their impact not only on mummification rates but also on fecundity of non-mummified aphids that have been stung by wasps as detected by a qPCR assay to detect parasitoid DNA, with experiments simulating a single parasitoid attack event. *Regiella* produced a sharp decrease in mummification rates of their native and transinfected hosts, but aphids with *Regiella* that were stung (successful oviposition confirmed by molecular analysis) and not mummified showed low reproduction. The combination of these effects meant we did not detect major differences in the population size of infected and uninfected aphids. These results highlight the importance of considering multi-trait effects when assessing the impacts of endosymbiont infections and predicting the outcome of endosymbiont–host–parasitoid interactions.

## 1. Introduction

Aphids (Aphididae) are small, soft-bodied insects, which include some of the most agriculturally important pests globally [1]. They can build up rapidly through parthenogenetic reproduction and cause substantial losses in yield across many industries [2]. Ecologically, aphids function as major herbivores, supporting microbial and ant communities and providing key prey for many predators such as lady beetles, hoverflies, lacewings as well as parasitoid wasps that are typically considered key biocontrol agents [1,3]. Aphids are also known to carry bacterial symbionts, which can include primary endosymbionts always found in a species (mainly buchnera, essential to aphids for providing nutrition) and secondary endosymbionts whose frequency can vary within aphid populations. Secondary endosymbionts can have a range of phenotypic effects on their hosts, influencing host interactions with viruses, fungi and parasitoids as well as affecting host fitness [4,5].

Parasitoid wasps lay their eggs inside the aphid's body via their ovipositor, where wasp larvae develop by feeding on the aphid's tissues, eventually killing the host and forming a mummy before an adult wasp emerges. Parasitoid development is influenced by external factors including temperature, host plant and interference from other parasitoids or predators [6,7]. In addition, internal factors including aphid genotype and bacterial endosymbionts present in aphid hosts can be important, impacting wasp development and emergence from the host [8,9]. Endosymbiont impacts, including those associated with some strains of *Hamiltonella defensa* and *Regiella insecticola*, are often scored through their effects on mummification rates that reflect successful wasp larval development [8–10]. Mummification rates can be variable depending on aphid genotype, endosymbiont strain and parasitoid wasp species [11,12], though protection from mummification is expected to affect aphid population size [13]. However, aphid host (and ultimately wasp) population impacts will depend on other factors as well ( figure 1), which may include post mummification emergence and impacts of other fitness traits including reproduction in surviving aphids [14,15].

**Figure 1.**
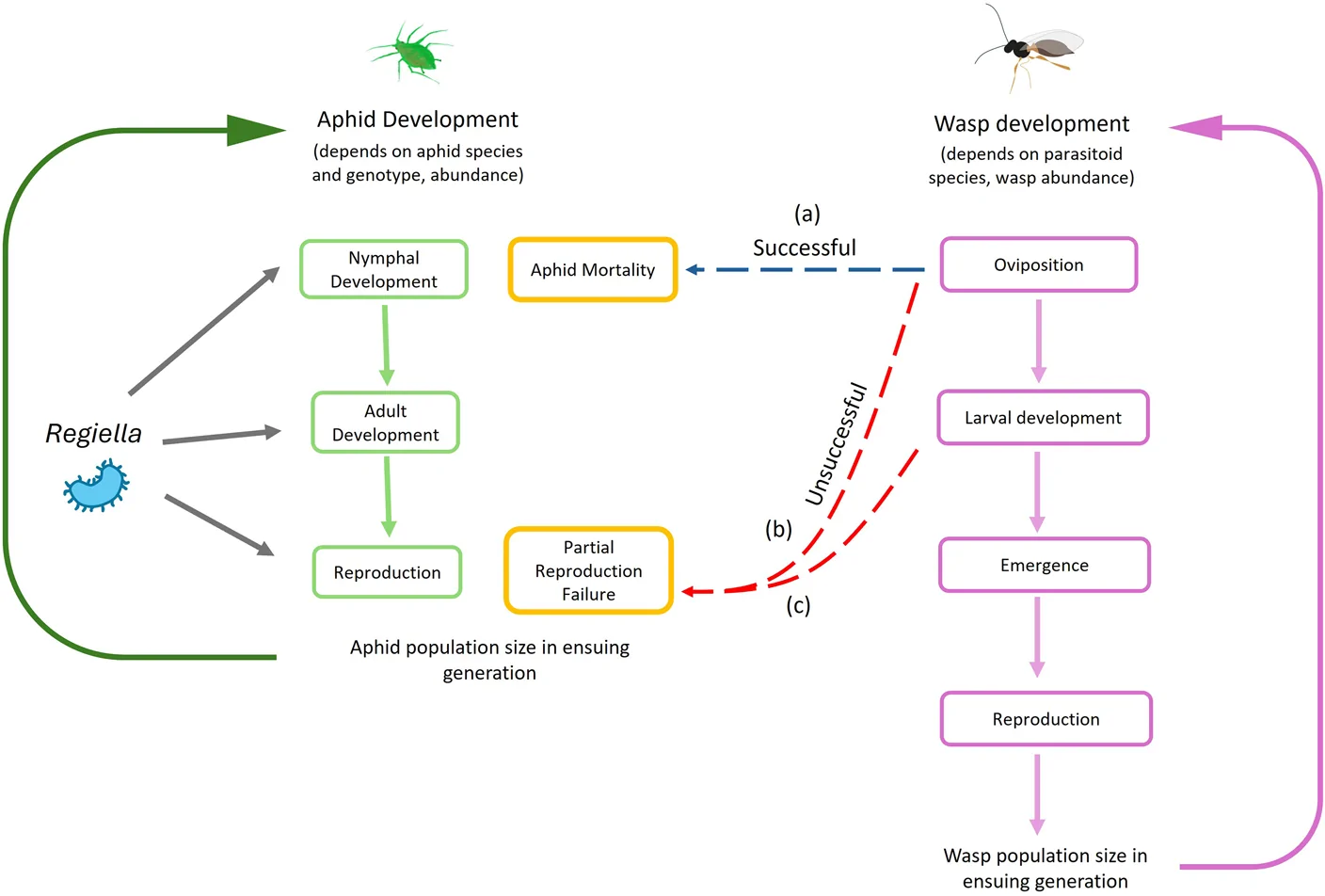
Potential host–parasitoid–*Regiella* interactions influencing aphid and wasp population growth. Individual development of wasp aphid (green), wasp (pink) and impacts on aphid development when interacting with wasp (orange). The arrows link to these interaction impacts between the wasp and the aphids, where there is (a) a successful oviposition event (dashed blue arrow) or two unsuccessful interactions impacting both wasp and aphid development (dashed red arrows). (b) Represents unsuccessful oviposition but a stinging event that can still impact aphid development, while (c) represents unsuccessful wasp larval development following oviposition. The first event leads to aphid mortality, while the second two events cause partial reproduction failure of the aphids, with both types of processes potentially influenced by *Regiella.*

Here, we are focused on the interaction between aphids and the symbiont *R. insecticola*. In 2005, a single population of *R. insecticola* was discovered in an Australian *Myzus persicae* [9], which reduced parasitoid mummification rates in its native host and when transinfected into another aphid species, *Aphis fabae*. The infection had no other discernible costs or benefits on its aphid host in the absence of parasitoids [12,16], although *Regiella* in aphids is also known to influence interactions with viruses and fungal pathogens in the host as well as host plant effects on fitness [17–19]. In the case of the *R. insecticola* strain identified as influencing mummification, there was also a reduction in fecundity in an infected aphid clone that survived parasitism by the wasp, *Aphidius colemani* [20]. A fitness reduction in surviving aphids was similarly noted in *A. fabae* harbouring two strains of *Hamiltonella* and exposed to *A. colemani* [15], though not when exposed to the wasp *Lysiphlebus fabarum* (an important parasitoid of *A. fabae*). Such effects beyond mummification may help explain why *R. insecticola* is not more common across Australia and elsewhere [21,22] and more generally help explain why changes in the frequency of secondary endosymbionts like *Hamiltonella* reducing mummification do not closely track parasitoid incidence (e.g. [23], p. 71).

We expand on this earlier work by focusing on another natural infection of *M. persicae* harbouring *R. insecticola* from Australia and transinfected into two other pest aphid species significant within broad acre crops, namely *Rhopalosiphum padi* [24] and *Diuraphis noxia* [25]. The infection is almost identical in terms of its DNA sequence to the original line from Vorburger *et al.* [9], which also sharply decreases mummification rate, but was present in a different *M. persicae* clone [26]. Focusing on the most common parasitoid wasp, *Diaeretiella rapae*, attacking *M. persicae* in Australia [27] as well as on *A. colemani*, we assessed the impact of *Regiella* not only on mummification rates in the native and transinfected hosts but also on post-mummification survival and aphid reproductive output. Contrary to expectations, there was little impact of the endosymbiont on aphid population size. Using DNA markers to confirm presence of parasitoid DNA in developing aphids carrying the *Regiella* infection, we showed a sharp reduction in aphid reproduction even when the infected aphid survived the oviposition event. These findings highlight the challenges in assessing ‘protective’ effects of endosymbionts in aphids when going beyond mummification.

## 2. Methods

### 2.1. Plant preparation

Bok choy (*Brassica rapa* subsp. *chinensis* var. baby bok) and wheat (*Triticum aestivum* cv. Trojan) were used in all aphid maintenance and experiments. Bok choy was sown into pots (70 × 70 × 160 mm) filled with potting mix (Osmocote Premium, Scotts Australia Pty Ltd., Victoria), with two seeds per pot. Bok choy leaves were used after 4–5 weeks old for culturing *M. persicae*. Wheat used for maintaining *D. noxia* and *R. padi* was sown under a hydroponic system setting in a mix of vermiculite (Exfoliators (Aust) Pty Ltd., Victoria) and perlite (Exfoliators (Aust) Pty Ltd., Victoria; 75 : 25) supported with liquid nutrients (2 ml Green Planet Dual Fuel solution: 1 l water). The wheat seedlings were left to develop for two weeks before use (Zadoks Growth Stage GS12).

Plant material used in the experiments were sown into pots (70 mm diameter × 70 mm height) using the same potting mix. Wheat plants were used 8 days post sowing (Zadoks Growth Stage GS10-11), while bok choy was used 14 days post sowing. All plant material was grown in a CT room set to 20°C ± 1°C with growth lights (TS1000 150W Dimmable Full Spectrum LED, Mars Hydro Australia) set to 16L : 8D.

### 2.2. Aphid cultures

A clone of *M. persicae* was collected in 2023 (Burnley, Victoria) from capsicum (*Capsicum annuum*) harbouring a natural *R. insecticola* strain [24]*.* This line was cured of *R. insecticola* [26] to create *Regiella*-infected and uninfected lines of the same clone. For both *R. padi* and *D. noxia*, the uninfected lines were collected as naturally uninfected clones, for which we used microinjection to derive the corresponding *Regiella*-infected lines, as described elsewhere [24,25]. All original aphid lines were established using offspring from a single isolated female to ensure clonality. The status of *R. insecticola* in each line was determined with qPCR before use in the experiments and lines were also shown to be free of other secondary endosymbionts [21].

Each aphid line was built up using cut host plants (bok choy leaves including petiole for *M. persicae*, whole wheat for *R. padi* and *D. noxia*) with a cup culturing method [24] for two weeks at 20°C, 16L : 8D. Nymphs from these colonies were used for adult age matching where the youngest nymphs (1–2 days old) were added to fresh plant material with the method described above, and then kept at 20°C, 16L : 8D for 9 days. After this period, apterous adults from the plants were used in the experiments.

### 2.3. Arasitoid preparation

*D. rapae* and *A. colemani* were used in all experiments, given their wide distribution and commercial availability within Australia [28]. Mummies of each parasitoid were supplied by Biological Services (Loxton, South Australia). These were separated into groups of 100 mummies and placed into plastic vials (20 mm diameter × 100 mm high). The vials were sealed with a mesh cap (160 µm). A drop of honey was placed on top of the mesh to feed the emerging wasps food source. Emergence of wasps was monitored, and once roughly 50% had emerged, they were left for a further 24 hours to allow for mating before being used in experiments. Batches of wasps were anaesthetized for short periods (less than 5 minutes) with CO_2_ and sexed under a stereo microscope (Olympus SZ40, magnification 0.67×) to separate out females through the following features:

*Diaeretiella rapae*: Female antennae with 13–15 segments and ovipositor. Male with 16–17 segments [29].

*Aphidius colemani*: Female antennae with 15 or 16 segments and ovipositor. Male with 16 segments [30].

Individual females were then placed into Eppendorf tubes (1.7 ml) with a drop of honey in the lid and allowed to recover for 24 hours before use.

### 2.4. Parasitism assay: impact of endosymbionts on parasitoid performance

To determine the effect of *R. insecticola* on parasitism, *M. persicae*, *R. padi* or *D. noxia* lines were exposed to two parasitoid species, *D. rapae* and *A. colemani*. Once host plants had developed (as described above), the plant material was passed through a hole (7 mm diameter) in a base of a 150 ml plastic cup. The cup was pressed into the soil, and the hole was sealed with cotton wool. Plants were left for 1 day before aphid introduction. Each plant had two apterous aphids (either *Regiella*-infected or uninfected) added to the host plant. The cups were sealed by adding another cup on top with the base of this cup replaced with mesh; a strip of Parafilm was placed around the two cups to seal them together. The aphids and plants were haphazardly positioned in a 20°C CT room supported by lights set at 16L : 8D and left for 24 hours. After this period, the adult aphids were removed and stored in ethanol for confirming infection status. Any replicate that had only one aphid (infected or uninfected) were excluded. This was to confirm an absence of contamination between the infected and uninfected populations. Nymphs were counted (average 11 ± 4 nymphs per cup) and allowed to develop for another 48 hours (reaching 2nd–3rd instar stages) to produce similar-aged cohorts.

Four treatments were set up separately but in the same way for each species. These treatments were (i) *Regiella* infected aphids with a parasitoid, (ii) *Regiella* uninfected aphids with a parasitoid, (iii) *Regiella* infected aphids without a parasitoid, and (iv) *Regiella* uninfected aphids without a parasitoid.

A single female parasitoid was introduced into the inverted cup with aphid nymphs, set up by removing the Parafilm holding the cups together and placing an opened Eppendorf tube with the wasp into the bottom cup before resealing. All replicates were stored in the same 20°C CT room and left for 48 hours. Each pot was haphazardly positioned. After 48 hours, the wasp and Eppendorf tube were removed.

Populations were left for 48 hours post parasitoid removal and scored every 24 hours for mortality of aphids unrelated to mummification. Mummies could be seen 6 days after parasitoid removal and continued to be scored daily until 48 hours had passed without new mummies appearing or until all aphids had become mummies. The mummified aphids were removed and placed in plastic vials (20 mm diameter × 100 mm high), one vial per replicate, and sealed with a mesh cap. Presence of wasp emergence was scored from 6 days after the first mummy was isolated. Once the wasps emerged, they were counted and removed. The experiment involved 35–40 replicates per wasp species, aphid species and aphid infection status. In addition, 10–15 control plants were set up per treatment without parasitoids. Each aphid species was tested separately given that the priority in these experiments was to compare treatments rather than aphid species.

We obtained four different measurements from our experiment: (i) mortality of aphids not related to mummification, (ii) proportion of aphids that survived, (iii) relative mummification rate (mummification/available nymph population = proportion of aphids mummified by the wasps, assuming that these survived), and (iv) wasp emergence success (number of wasps emerged/total mummies).

### 2.5. Population level effects: impact of endosymbiont–parasitoid interactions on aphid reproduction

We examined population effects of *Regiella* harboured by both *M. persicae* and *D. noxia* to assess development and recovery after the single parasitoid exposure event described in the previous section. After mummification scoring had been completed (in the Parasitism assay described above), all surviving adults were left for an additional two or three weeks. This design reflects the situation where there is a single parasitism event, and populations have the potential to recover after the event. Owing to the destructive nature of this assessment, half the replicates were counted at week 2 and the other half at week 3, although controls (never exposed to wasps) were only measured at week 2 and only for *M. persicae*, because there were fewer replicates available for this treatment. To collect aphids, plant material was cut at the base and placed in a plastic cup (150 ml) with 40 ml of 100% ethanol with the assistance of a fan shaped paint brush. The cup was then sealed with Parafilm and stored at −20°C. The content of each cup was filtered through a funnel lined with paper towel to collect the aphids and plant material. The aphids were brushed off the plant material and paper towel into a 30 mm diameter Petri dish with 100% ethanol and separated into four sections to facilitate counting using a stereo microscope (Olympus SZ40, magnification 0.67×).

### 2.6. Individual reproduction assay: impact of endosymbiont–parasitoid interactions after parasitism

Because of an unexpected similarity in population recovery in *Regiella-*infected and uninfected lines (see §3), we conducted a separate experiment where we followed the development and reproduction of aphids harbouring *R. insecticola* exposed to the parasitoid. To determine the impact of parasitism in this experiment, we tested: (i) the fecundity of aphids after they had been exposed to the parasitoid, and (ii) the presence of parasitoid DNA in these surviving aphids via PCR. The method of wasp exposure and parasitism followed the procedure described above (‘Parasitism assay: impact of endosymbionts on parasitoid performance’) and was carried out with *M. persicae* and *D. noxia*.

Six replicate plants were set up for *M. persicae* per wasp species and four replicate plants for *D. noxia* (each with 9–13 nymphs per plant). Four controls were also set up per aphid species in the same way but these aphids were not exposed to parasitoids. For plants exposed to wasps, a single female was left in the container for 48 hours. After parasitoids were removed, the aphids were left for 48 hours before live aphids were individually placed into 35-mm petri dishes with 1% agar and discs of their respective host plant. The 48-hour period after wasp removal allowed aphids to recover from wasp exposure and to develop further before they were handled. For *M. persicae*, bok choy leaf discs were cut into discs (25 mm diameter), while for *D. noxia*, wheat leaves were cut into pieces 25 mm long by 5 mm wide. Each day, these aphids were checked for development until they became adults and produced nymphs. Fecundity was recorded every 24 hours for 12 days once the controls (aphids not exposed to wasps) started reproducing. Once reproduction was finished, surviving aphids were stored in 100% ethanol and the presence of parasitoid DNA was tested in a subset of aphids using qPCR (see §2.8). For *M. persicae*, 32 aphids exposed to *D. rapae* were screened, as were 19 exposed to *A. colemani* and 10 controls. For *D. noxia*, screening was undertaken for 10 aphids from *D. rapae*, 18 from *A. colemani* and 10 from the controls.

### 2.7. Stinging assay: impact of observed stinging events on aphid reproduction at the individual level

In the previous ‘individual reproduction assay’ experiments, wasp oviposition events were assumed but not directly observed. A preliminary molecular analysis suggested that there was a negative association between the presence of parasitoid DNA detected in aphids with their reproductive output (supplementary material, figure S1). However, without direct observations of stinging, we could not verify that detected parasitoid DNA was the result of an attempted oviposition event.

We therefore conducted a follow-up experiment to directly test the association between stinging events by parasitoids and reduced aphid fecundity. The overall approach involved (i) observing the parasitoid stinging event, (ii) scoring reproduction of individuals stung or exposed to wasps, and (iii) confirming stinging by testing for the presence of parasitoid DNA. This experiment used *M. persicae* and the wasp species *D. rapae*.

Arenas were set up as Petri dishes (60 × 15 mm) with cut leaf discs (25 mm diameter) of bok choy set in a 1% agar water solution. Ten 3-day-old nymphs of *M. persicae* reared using the methods described previously from either the *Regiella*-infected or uninfected line were added to each arena. The aphids were left for 1 hour to settle before wasp exposure. There were six arenas for the *Regiella*-infected or uninfected line exposed to the wasp, as well as three arenas per line without exposure to a wasp.

For the wasp treatments, a single mated *D*. *rapae* female wasp was introduced into the arena. The arena was monitored to determine if stinging took place. A stinging event was considered successful if the ovipositor made contact with an aphid for 2 seconds. The stung aphid was then removed immediately and placed onto a fresh bok choy leaf disc set into a 35-mm Petri dish with 1% agar. This continued until 5 out of the 10 aphids had been stung. The wasp was then removed, and the non-stung aphids were placed onto their own bok choy leaves in Petri dishes. This then resulted in three separate classes of aphids: (i) stung, (ii) not stung, and (iii) controls without exposure to a wasp. Individual aphids from the treatments were placed in a 20°C CT room haphazardly.

All aphids were scored every 24 hours post wasp removal for early mortality (dead before mummification), mummification and first signs of reproduction. Once controls started reproducing, this was considered day 1 and all treatments were scored every 24 hours subsequently for nymph production, mortality during reproduction and delayed mummification, until day 9 when all remaining live aphids were removed and stored in 100% ethanol for scoring *Regiella* infection and the presence of parasitoid DNA.

### 2.8. Molecular detection of endosymbiont and parasitoid DNA

To confirm the presence or absence of *Regiella*, qPCR assays were used to measure their density relative to a host gene [31,32]. In the experiments, adults used to produce the treatment aphids were checked and confirmed as positive with a *Regiella C*_p_ value ≤ 30 and *T*_m_ value within the range of positive controls (87.5–88.3). Aphids were considered negative if *C*_p_ values were not obtained and *T*_m_ values were not within the range of positive controls. Aphids checked for *Regiella* presence were all confirmed infected.

To detect the presence of parasitoid wasp DNA in aphids, we developed a qPCR assay using the 16S-F (CGCCGTTTTATCAAAAACATGT) and 16S-Rspe (TCTAWAGGGTCTTCTCGTCT) primers outlined elsewhere [33]. The assay detected parasitoid wasp infection based on the melting temperature [32] of a 210bp 16S-Rspe amplicon. High-resolution melt analysis on the Roche LightCycler 480 system produced a *T*_m_ range from approximately 76.55–76.74 for *A. colemani* and 77.11–77.26 for *D. rapae*. The quality of the assay was verified using a standard curve analysis which utilizes a range of amplification success measures to test efficiency and optimize primers [34]. Amplification accuracy of the primers was also verified by Sanger sequencing PCR products for two *A. colemani* and one *D. rapae* sample using Macrogen (South Korea). The chromatograms were checked in Geneious version R9.1.8, http://geneious.com [35], and the sequences were confirmed to be from *A. colemani* or *D. rapae.*

Samples were extracted using a 5% Chelex solution (Bio-Rad Laboratories, Gladesville, NSW Australia; Cat. No. 1421253EDU; w/v in distilled water) tube-based method whereby a sample was added to a 1.5 µl tube with 250 µl of 5% Chelex solution, two 3-mm glass beads and 2.5 µl of Proteinase K (20 mg ml^−1^; Meridian Bioscience, London, UK). Samples were homogenized in a Tissue Lyser (QIAGEN, Clayton, VIC, Australia), then incubated at 65°C for 60 minutes, followed by 90°C for 10 minutes. DNA was diluted to 1 in 5 for downstream investigations.

qPCR amplifications were carried out on a Roche LightCycler 480 system in a 10 µl reaction volume (reagent volumes as per Lee *et al.* [31]; supplementary material), except for the IMMOLASE DNA polymerase which was used at 3× the amount). The qPCR amplification conditions were as follows: 10 min at 95°C; 40 cycles of 1 min at 94°C, 1 min at 62°C, 90 s at 72°C and a final extension for 7 min at 72°C. We attempted to shorten the programme duration in line with typical qPCR programmes; however, this reduced the quality of the *T*_m_ curves, so we opted to retain the longer programme.

While we were able to determine the presence and absence of parasitoid wasp DNA using this protocol by confirming a *T*_m_ peak at the target temperature, small secondary peaks were often present which meant we were unable to use the cycle number at detection threshold (*C*_p_) for relative quantification of DNA as has been done elsewhere (e.g. [36], p. 508). Indeed, secondary peaks and low DNA yield often meant that results were unclear from the *T*_m_ value alone and that *T*_m_ peaks needed to be visualized alongside those of positive controls to be certain about infection status.

### 2.9. Statistics

All statistical analyses and visualizations were conducted in R. The *data.table* [37], *readxl* [38] and *tidyverse* [39] packages were used for data wrangling. Graphs were made using *ggplot2* [40] and *ggpubr* [41]. Post hoc pairwise tests were performed with a combination of the *emmeans*, *multcomp* and *multcompView* packages.

#### 2.9.1. Parasitism assay

We tested the hypotheses that mortality in each aphid species was dependent on its endosymbiont infection status and the wasp species attacking it. Here, we treated mortality in the broadest sense given that we are interested in overall endosymbiont effects: aphids that died early (but did not become mummies), or those that became mummies, were pooled. Models were fitted for each aphid species separately, following a binomial error distribution, taking the form: Mortality = Endosymbiont + Wasp + Endosymbiont : Wasp.

Here, the response ‘Mortality’ is the mortality for each replicate represented by counts of dead and alive aphids. ‘Endosymbiont’ is the categorical fixed effect of infection status, with levels including uninfected and infected by *Regiella*. ‘Wasp’ is the categorical fixed effect of parasitoid exposure, with levels including the unexposed control, exposure to *A. colemani* and exposure to *D. rapae*. Models were fitted with the *glmmTMB* function [42], with significance of terms tested using the *Anova* function from the base R *car* package.

The same model structure was used to test the hypothesis that relative mummification in each aphid species was dependent on its endosymbiont infection status and the wasp species attacking it. Here, we consider the relative parasitism as the number of aphids that survive early mortality and that go on to form mummies. These data came were fitted for each aphid species separately, as above.

We tested the hypotheses that successful parasitism in each aphid species was dependent on its endosymbiont infection status and the wasp species attacking it. For parasitism to be successful, a wasp must oviposit into an aphid, have its offspring complete its developmental cycle and finally emerge as an adult wasp. We subset our data to include only those aphids that incurred mortality and designated them to three categorical outcomes: aphids that died early before mummification, successful mummification but no wasp emergence and successful wasp emergence. These categories can be seen as ordinal responses because the probability of achieving successful parasitism (emergence) is dependent on the cumulative mortality of the wasp. Models were fitted for each aphid species separately, taking the form:

$$\begin{array}{r} Outcome=Endosymbiont+Wasp+Endosymbiont:Wasp+Replicate;and\\ Outcome=Endosymbiont+Wasp+Replicate. \end{array}$$

Here, the response ‘Outcome’ is the log-odds that the observed outcome for an individual aphid in a replicate is equal or less than another possible outcome (death before mummification < mummification but no emergent wasp < successful wasp emergence). ‘Endosymbiont’ is the categorical fixed effect of infection status, with levels including uninfected and infected by *Regiella*. ‘Wasp’ is the categorical fixed effect of parasitoid exposure, with levels including the unexposed control and exposure to *D. rapae*. ‘Replicate’ is the random intercept for each replicate group. Models were fit with the *clmm* function from the *ordinal* package [43]. Owing to issues of over-fitting, we reduced models to the simplest form following two criteria. First, if the parameter Hessian matrix of the full model was not positive definite, we used the additive model. Second, if model comparison supported equivalence between them, we used the additive model. Significance of model terms was tested using the *Anova* function.

#### 2.9.2. Population-level effects

We tested the hypothesis that the relative population-level increase in each aphid species was dependent on its endosymbiont infection status and the wasp species attacking it. We measured the population change: the total number of aphids at a census point minus the original number of aphids at the beginning of the experiment. We considered two census points: two and three weeks. Most models were fitted separately for each aphid species and census point, following a Gaussian error distribution, taking the form: Change = Endosymbiont + Wasp + Endosymbiont : Wasp.

For *M. persicae*, we did not have data for the control treatment at the week 3 census point, so instead fitted the model: Change = Endosymbiont + Wasp.

Here, the response ‘Change’ is the change in the number of aphids in a replicate population. ‘Endosymbiont’ is the categorical fixed effect of infection status, with levels including uninfected and infected by *Regiella*. ‘Wasp’ is the categorical fixed effect of parasitoid exposure, with levels including the unexposed control, exposure to *A. colemani* and exposure to *D. rapae*. Population-level increase models were fitted using the *lm* function, with significance tested using the *aov* function, from base R.

#### 2.9.3. Individual reproduction assay

We tested the hypothesis that fecundity of *Regiella*-infected aphids was dependent on wasp attack. We measured the number of nymphs produced by aphids that originally came from the same plant. Models were fitted for each aphid separately, following a negative binomial error distribution, taking the form: Fecundity = Parasitoid + Plant/Replicate.

Here, the response ‘Fecundity’ is the number of nymphs produced by a replicate individual aphid nested in a plant. ‘Parasitoid’ is the categorical fixed effect of parasitoid exposure, with levels including the unexposed control, exposure to *A. colemani* and exposure to *D. rapae*. ‘Replicate’ is the random intercept for replicate individual, nested in the effect of ‘Plant’, which is the random intercept for the plant. Models were fitted using the *glmmTMB* function with significance of terms tested using the *Anova* function.

#### 2.9.4. Stinging assay

For our final analysis, we tested the hypothesis that an observed stinging event was the cause of reduced fecundity of *Regiella-*infected aphids. We measured the total fecundity of aphids that were stung by a wasp and those that were not stung but still exposed to wasps. To be included in the model, an aphid needed to survive until the point of measurement (no early mortality or mummification). Our model was fit following a negative binomial distribution and took the form: Fecundity = Infection + Sting + Replicate.

Here, the response ‘Fecundity’ is the log-transformed number of nymphs produced by an individual aphid in a replicate. ‘Infection’ is the categorical fixed effect of infection status, with levels including uninfected and infected by *Regiella*. ‘Sting’ is the categorical fixed effect of sting status, with levels including not stung and stung by *D. rapae*. ‘Replicate’ is the random intercept for each replicate group. Models were fitted using the *glmmTMB* function with significance of terms tested using the *Anova* function. Note: we could not fit the full interaction model because the design was not fully factorial (there were no surviving stung aphids that were uninfected with *Regiella*).

## 3. Results

### 3.1. Parasitism assay

Aphid mortality rates were low in controls with no effect of *Regiella* (figure 2). However, in the full analysis, mortality rates differed significantly with respect to *Regiella* infection status, wasp treatment and their interaction, across all three aphid species (supplementary material, tables S1 and S2). Within each species, aphids that were uninfected with *Regiella* consistently incurred more mortality relative to aphids that were infected with *Regiella* when exposed to wasp attack (‘Endosymbiont’ effect, df = 1: *D. noxia*, *X*^2^ = 254.5, *p* < 0.001; *M. persicae*, *X*^2^ = 385.8, *p* < 0.001; *R. padi*, *X*^2^ = 322.7, *p* < 0.001). However, even with *Regiella* infection, mortality within each species was still typically greater than that observed in the control treatments (‘Wasp’ effect, df = 2: *D. noxia*, *X*^2^ = 47.4, *p* < 0.001; *M. persicae*, *X*^2^ = 91.8, *p* < 0.001; *R. padi*, *X*^2^ = 225.2, *p* < 0.001). The interaction between endosymbiont infection and wasp treatment was significant for all aphid species (supplementary material, table S1). In *D. noxia*, *A. colemani* tended to kill fewer infected aphids, relative to *D. rapae*. In *M. persicae*, *A. colemani* and *D. rapae* tended to kill similar numbers of aphids. In *R. padi*, *A. colemani* consistently killed more aphids relative to *D. rapae* (figure 2).

**Figure 2.**
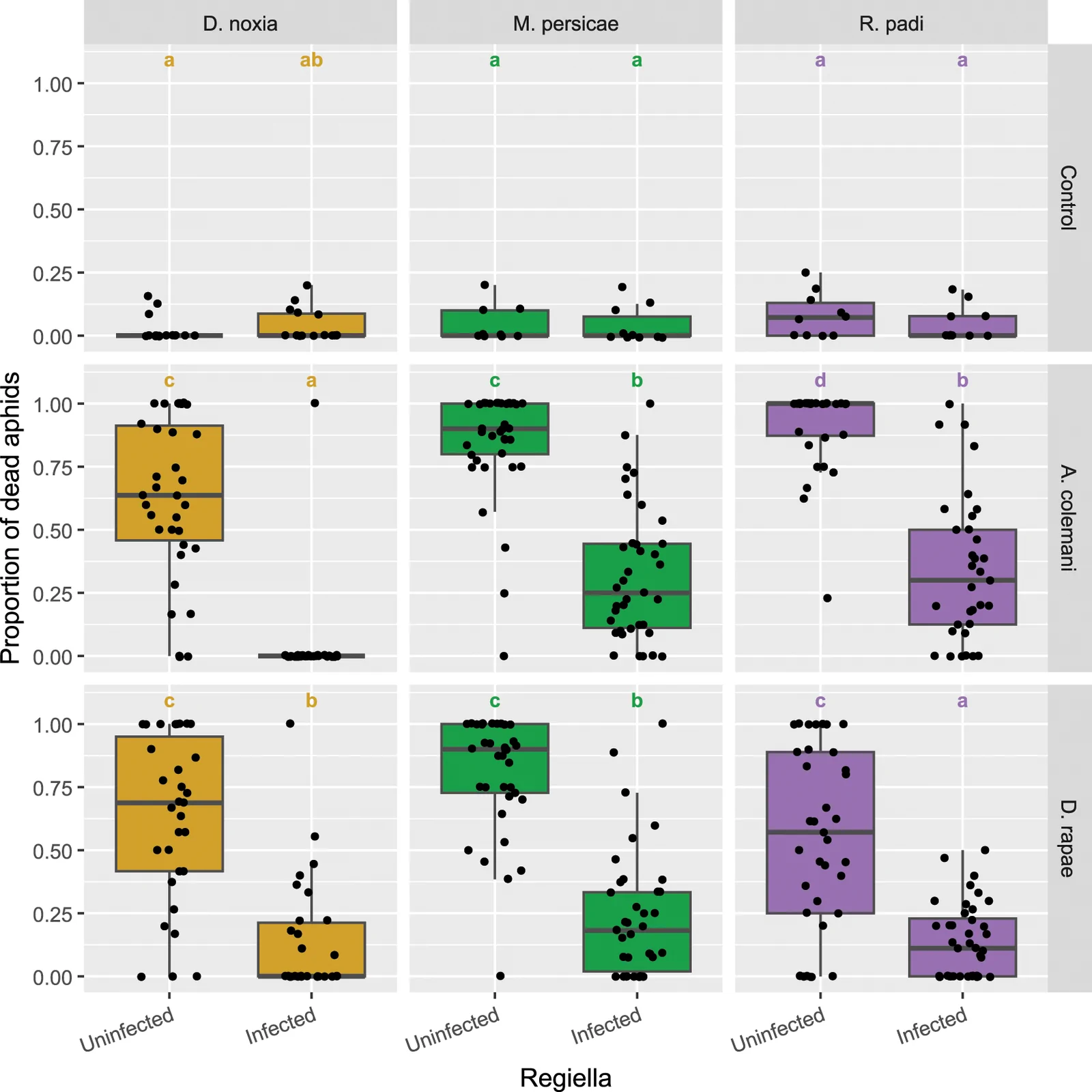
Boxplots of aphid mortality associated with parasitoids when aphids are uninfected or infected by *Regiella*. Each point represents an independent replicate. Each point represents an independent replicate. Letters indicate non-significant experimental groups, as evaluated through post-hoc tests; these should be compared down column only (within each aphid species).

Aphid mummification rates also differed significantly with respect to *Regiella* infection status for all three aphid species (supplementary material, tables S3 and S4). Within each species, aphids that were uninfected with *Regiella* became mummified more often relative to aphids that were infected with *Regiella* (figure 3; ‘Endosymbiont’ effect, df = 1: *D. noxia*, *X*^2^ = 60, *p* < 0.001; *M. persicae*, *X*^2^ = 160, *p* < 0.001; *R. padi*, *X*^2^ = 97, *p* < 0.001). Within aphid species, the main effect of the wasp treatment was only significant in *R. padi*, where *A. colemani* more frequently produced mummies relative to *D. rapae* (‘Wasp effect’, df = 2: *D. noxia*, *X*^2^ = 0.001, *p* = 0.99; *M. persicae*, *X*^2^ = 2.4, *p* = 0.49; *R. padi*, *X*^2^ = 28.7, *p* < 0.001). The interaction between endosymbiont status and wasp treatment was non-significant within all three aphid species (supplementary material, table S3).

**Figure 3.**
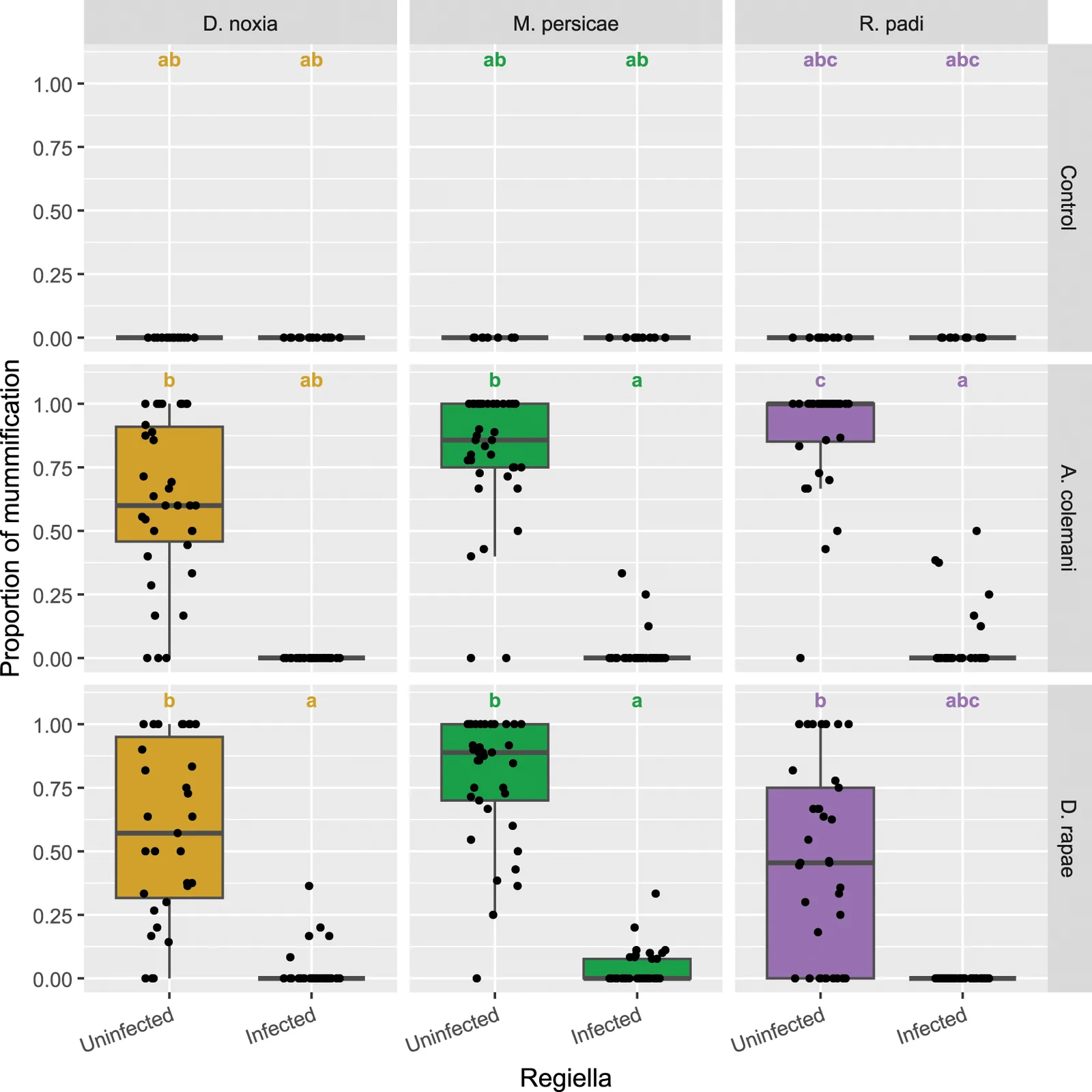
Boxplots of relative mummification (proportion of nymphs mummified) associated with parasitoids when aphids are uninfected or infected by *Regiella*. Each point represents an independent replicate. Letters indicate non-significant experimental groups, as evaluated through post-hoc tests; these should be compared down column only (within each aphid species).

Within each species, wasp emergence success was most simply explained by the additive model, including just the additive effects of *Regiella* infection status and wasp species (supplementary material, tables S5 and S6). Aphids that were infected with *Regiella* were less probably to lead to successful emergence of wasps across all three aphid species (‘Endoymbion’ effect, df = 1: *D. noxia*, *X*^2^ = 22.8, *p* < 0.001; *M. persicae*, *X*^2^ = 136.9, *p* < 0.001; *R. padi*, *X*^2^ = 71.5, *p* < 0.001). In *D. noxia*, there was no significant difference in outcomes if the aphid was parasitized by *D. rapae*, relative to *A. colemani* (‘Wasp’ effect, *X*^2^ = 1.3, *p* = 0.25). However, in *R. padi*, attack by *D. rapae* was less probably to lead to successful emergence of wasps (‘Wasp’ effect, *X*^2^ = 6.7, *p* = 0.01), while the opposite trend was apparent for *M. persicae* (‘Wasp’ effect, *X*^2^ = 11.5, *p* = 0.001; figure 4).

**Figure 4.**
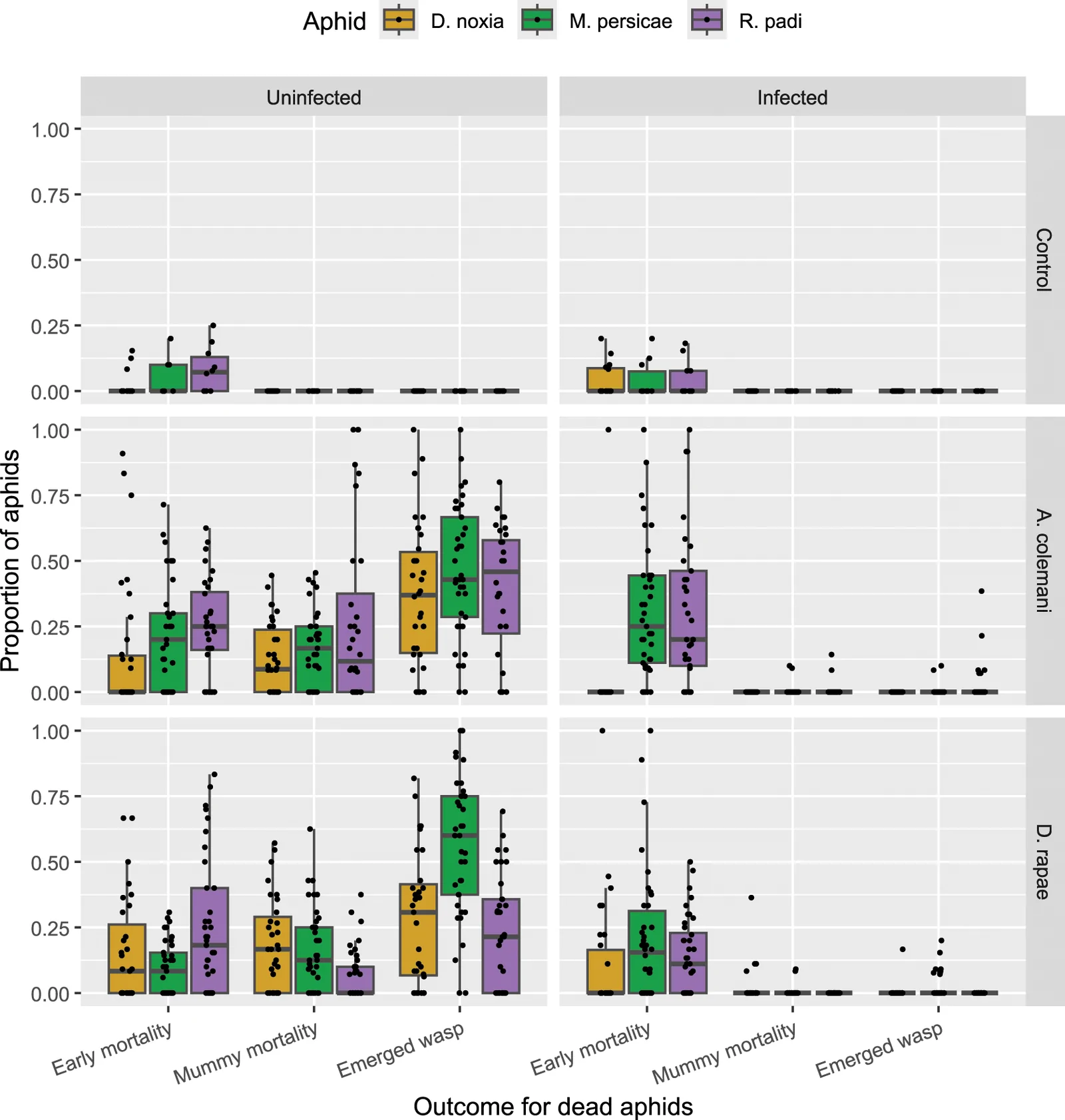
Boxplots of outcome of dead aphids at different developmental stages after exposure to wasps. Aphid outcomes included: death prior to mummification (early mortality), mummification without production of an adult wasp, mummification with a successfully emerged adult wasp. Each point represents an independent replicate.

### 3.2. Population-level effects

Population changes in aphid number differed significantly with respect to wasp exposure treatment across aphid species and census points (figure 5; supplementary material, tables S7 and S8; ‘Wasp’ effect: *D. noxia* week 2, F_2,65_ = 35.7, *p* < 0.001; *D. noxia* week 3, F_2,58_ = 11.9, *p* < 0.001; *M. persicae* week 2, F_2,84_ = 55.9, *p* < 0.001; *M. persicae* week 3, F_1,61_ = 0.49, *p* = 0.49)*.* Surprisingly, there was a non-significant main effect of *Regiella* infection status within each species: uninfected and infected aphids exhibited a similar population changes (‘Endosymbiont’ effect: *D. noxia* week 2, F_1,65_ = 0.14, *p* = 0.70; *D. noxia* week 3, F_1,58_ = 1.4, *p* = 0.23; *M. persicae* week 2, F_1,84_ = 0.38, *p* = 0.53; *M. persicae* week 3, F_1,61_ = 0.66, *p* = 0.42). There was only a significant interaction term for *D. noxia* at the week 3 census point (‘Endosymbiont : Wasp’ effect: F_2,58_ = 5.4, *p* = 0.007; figure 5). While we did not have control treatment data for *M. persicae* in week 3, the population change was comparable across the *Regiella* infected and uninfected lines and wasp species (figure 5).

**Figure 5.**
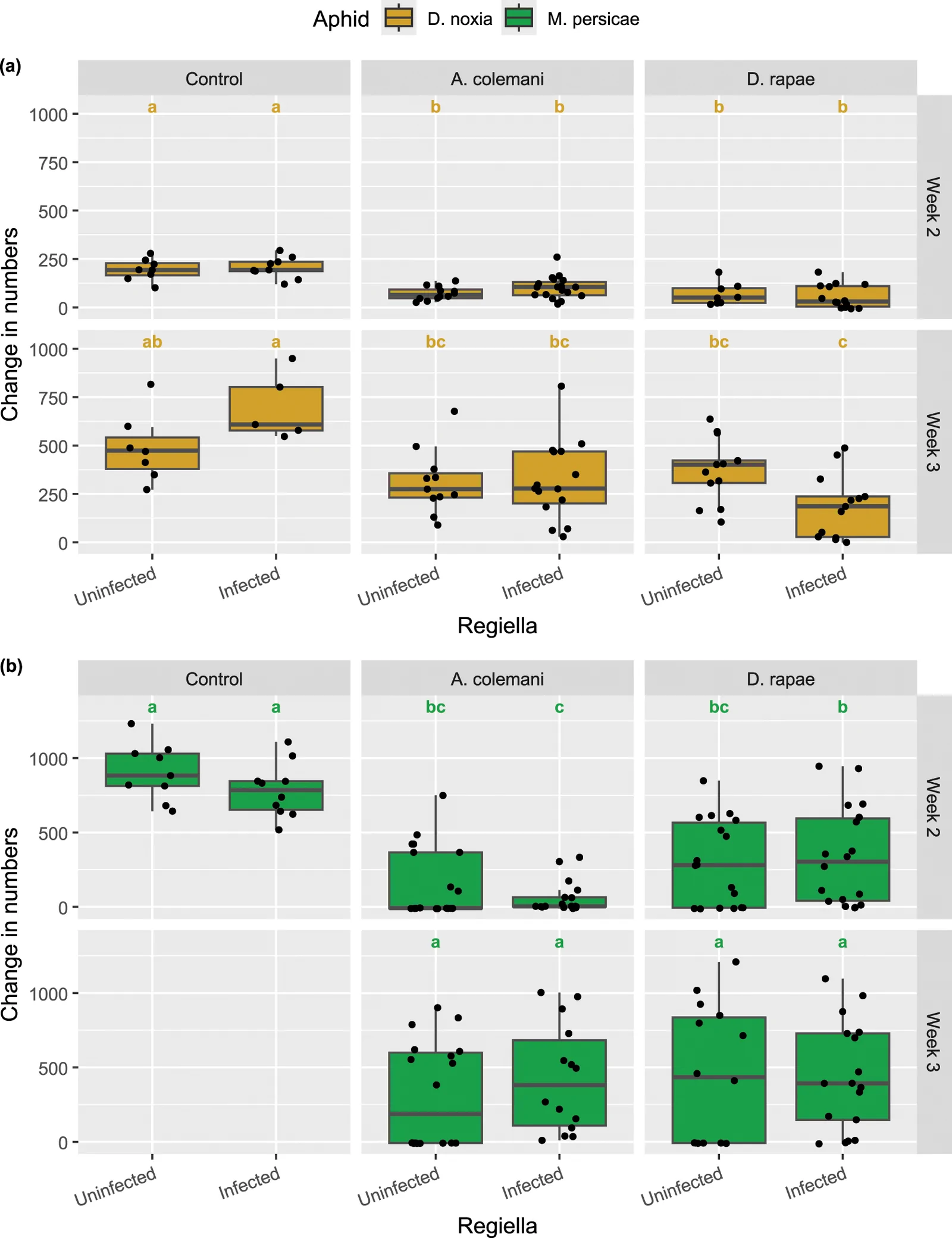
Boxplots of population recovery post wasp exposure at census points weeks 2 and 3. Represented by fold-increase in aphid numbers from the start of the experiment. Each point represents an independent replicate. (a) *D. noxia*. (b) *M. persicae*. Letters indicate non-significant experimental groups, as evaluated through post-hoc tests; these should be compared across rows only (within each week).

### 3.3. Individual reproduction assay

Nymph production in aphids infected with *Regiella* was significantly affected by wasp treatment within each aphid species (supplementary material, table S9). In *D. noxia*, treatments differed significantly (‘Wasp’ effect: *χ*^2^ = 20.8, df = 2, *p* < 0.001), and both *A. colemani* and *D. rapae* reduced nymph production relative to the control, while there was no significant difference between wasp species (figure 6). In *M. persicae*, treatments also differed (‘Wasp’ effect: *χ*^2^ = 44.5, df = 2, *p* < 0.001); both wasps reduced nymph production relative to the control, and *A. colemani* reduced the nymph production more than *D. rapae* (figure 6).

**Figure 6.**
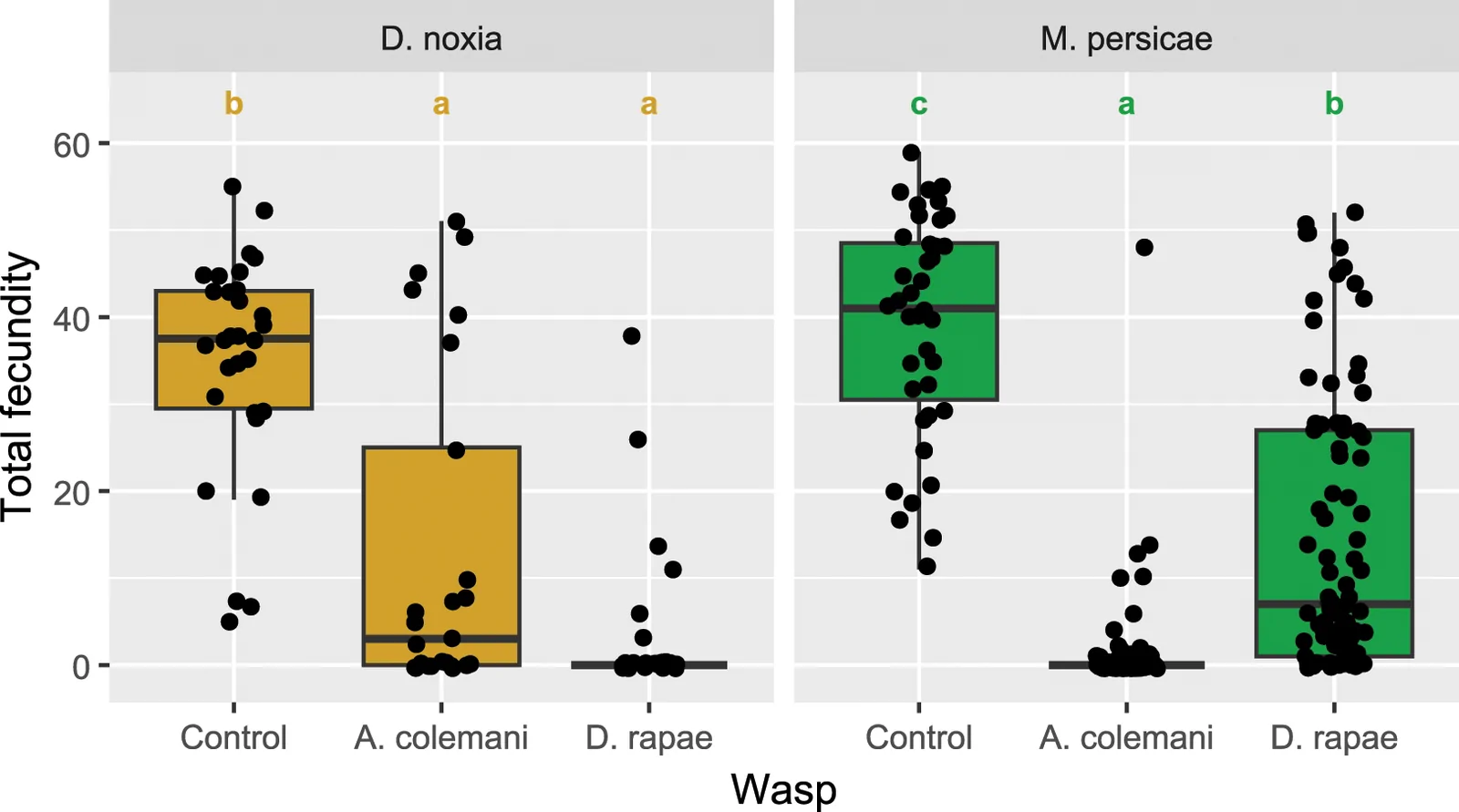
Impact of parasitoid wasps on individual-level reproduction of *Regiella*-infected aphids (*D. noxia* and *M. persicae*). Each point represents an individual aphid. Letters indicate non-significant experimental groups, as evaluated through post-hoc tests; these should be compared within each aphid species.

### 3.4. Stinging assay

Nymph production of *M. persicae* was significantly impacted by stinging treatment (‘Sting’ effect: *χ*^2^ = 92.1, df = 2, *p* < 0.001), irrespective of whether they were uninfected or infected with *Regiella* (‘Endosymbiont : Sting’ effect: *χ*^2^ = 0.55, df = 1, *p* = 0.46; supplementary material, table S10). Aphids that were exposed to, but not stung by, *D. rapae* tended to have lower fecundity relative to the control group, irrespective of their *Regiella* infection status (figure 7). This might suggest some influence of early life stress that carries over into the reproductive adult stage. Aphids that exposed to, and stung by, *D. rapae* had substantially reduced fecundity. In the case of *Regiella*-uninfected aphids, stinging led to mummification or death, so fecundity was zero across all replicate individuals (figure 7). Interestingly, stung *Regiella-*infected aphids also had very low fecundity: 57% of stung individuals produce 0 nymphs, despite carrying the putatively protective *Regiella* endosymbiont. These data indicate that stinging damages reproductive capacity, despite survival provided by the endosymbiont. Molecular diagnostics revealed strong concordance between observed stinging events and detection of parasitoid DNA in aphids (supplementary material, figure S2). Indeed, aphids with parasitoid DNA present produced fewer nymphs than those aphids in which parasitoid DNA was absent (supplementary material, figure S2).

**Figure 7.**
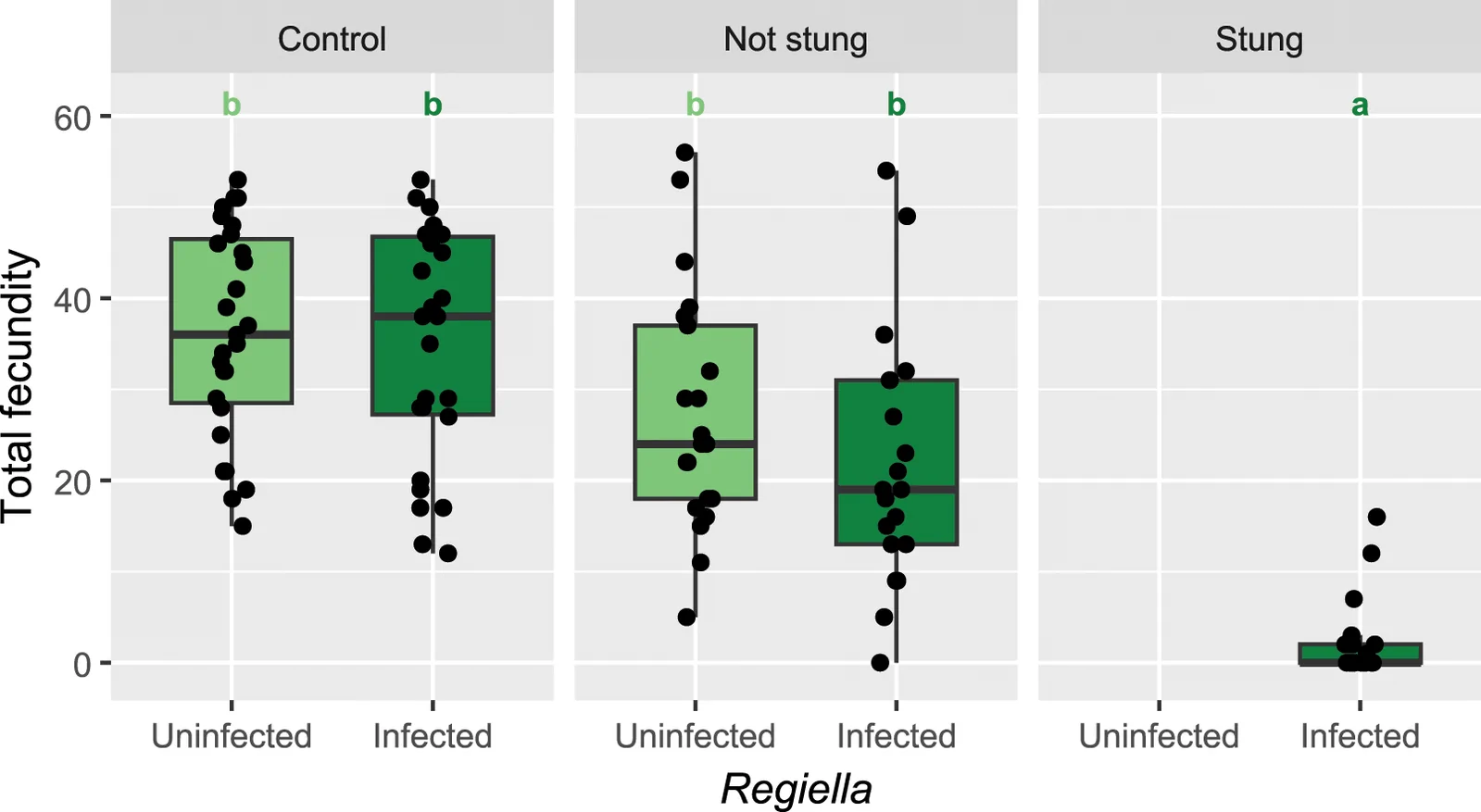
The effects of being stung or not stung by *D. rapae* on individual-level reproduction of *M. persicae* (uninfected and infected with *Regiella*). Each point represents an individual aphid. Letters indicate non-significant experimental groups, as evaluated through post hoc tests; these should be compared across all stinging status levels.

## 4. Discussion

In this study, we aimed to investigate interactions between two important parasitoids and the endosymbiont *R. insecticola* in a native host and two transinfected hosts, using infected and uninfected lines of one clone from each of these species to ensure that the same genetic background was involved. Building on other previous work both involving *Regiella* and *Hamiltonella* [15], we showed that parasitoid interactions with *Regiella* extended beyond detectable effects on mummification and to reproductive effects, with the result that there was little difference in aphid population size between infected and uninfected cultures by the end of the experiment. We interpret these results as reflecting substantial reproductive damage caused by parasitoid stinging, despite successful aphid survival, although toxicity from parasitoid venom or development of teratocytes could also be involved. We validated stinging events through molecular detection of parasitoid DNA and visual observation. These findings highlight the challenges of assessing parasitoid impacts by mummification rates alone, which has previously been noted in past work for the parasitoids used here [27,28].

The experimental design involved a single period of exposure to parasitoids rather than cross generation effects being also included. We imagine that this scenario is relevant to field situations where there might be a deliberate release of parasitoids or a short period favourable for parasitoids owing to an abundance of hosts. We note that both species of parasitoid tested here are available commercially (https://biologicalservices.com.au) and being released in some crops [44]. However, it is clearly also of interest to consider multigenerational effects particularly as interactions between hosts, parasitoids and endosymbionts is expected to alter wasp as well as aphid host (and *Regiella*) populations. Based solely on mummification as a ‘protection’ process, we might expect *Regiella* frequencies to increase over time, whereas aphid numbers (and also parasitoid numbers) should decrease relative to those in uninfected host populations. Field data would indicate that this dynamic involving endosymbiont protection (in one case linked solely to the presence of *Hamiltonella*) is not necessarily reflected in changes in endosymbiont frequency [23,45]. By contrast, with reproductive factors considered, aphid numbers might be expected to remain relatively steady until such a time that parasitoid numbers increase to the extent that they provide a high level of control. *Regiella* frequencies might then remain relatively similar across generations, though any minor advantage of *Regiella* may be amplified as parasitoid populations increase. However, a build-up of parasitoids could be curtailed in populations with *Regiella* given that wasp emergence from infected aphids was low.

Obviously, these patterns will be influenced by other factors including changes in the clonal composition of the aphid populations and the nature of the parasitoids. Clonal variation can influence parasitism rates in several ways, including directly influencing the susceptibility of aphids to parasitism (e.g. [46,47]) and in interacting with the effects of endosymbionts, as in *Sitobion* aphids [48]. In *M. persicae*, reproductive effects of stinging can depend on the clone [20]. Clonal–endosymbiont interactions have been detected not only in native infections but also in transinfected material as shown for morphological effects on *M. persicae* clones infected with *Rickettsiella* [32]. There are also many other issues around further interactions among the endosymbionts and parasitoids. For instance, do *Regiella* have impacts further downstream on wasp quality, as documented for *Regiella* in other aphids [49]? And do *Regiella* strains that fail to influence mummification provide any protection against sterilization, given that strain effects can be specific to particular aphid traits [14]?

The mechanisms that cause this protection are different between endosymbionts. Parasitism rates in aphids infected by *H. defensa* seem largely correlated to the presence of a bacteriophage APSE (*Acyrthosiphon pisum* secondary endosymbiont) [50]. When *Hamiltonella* does not contain APSE or is lost, then so is the protection [51,52]. APSE encodes homologs of bacterial eukaryote-targeting toxins from three protein families associated with arresting development of parasitoid larvae [10]. Protective effects of *Hamiltonella* can vary among parasitoids, as well as aphid biotypes [52] and variation among parasitoid responses has been linked to APSE toxin variability [53]. However, *R. insecticola* does not contain APSE. Instead, the protective strain contains five encoded categories of pathogenicity factors missing in the non-protective strain [54]. A combination of these genes may play an important role in parasitism reduction and may provide general protection against mummification regardless of the wasp parasitoid as observed here. However, the mechanisms that arrest larval development are poorly understood.

The process of damage and reproductive sterilization of aphids may depend on the secretion of venom or teratocyte proteins, impacting the development of host reproductive tissues [7]. The relevant mechanism involves ovariole development post oviposition by the parasitoid, particularly in young nymphs [55]. This would mean only those nymphs in early development suffer from sterilization when compared to an adult aphid being parasitized. Only a few cases of this phenomenon have been reported where an endosymbiont in surviving aphids affects development and reproduction [15,20]. Hafer-Hahmann & Vorburger [15] found such an impact on reproduction but this depended on endosymbiont strain and wasp species.

While research and review papers often refer to endosymbionts providing protection, perhaps this term detracts from the complex interactions involving aphids, endosymbionts and wasps. Each species in this interaction is expected to maximize its own fitness and in some ways the aphid might not benefit from the ‘protective’ endosymbiont except indirectly if parasitoid population expansion is curtailed. Instead of using terms like ‘protection’, it may be more appropriate to use more neutral terminology whereby all components of interactions can be considered within the context of eventual ecological and evolutionary outcomes [56]. Moreover, reference to protection is often based solely on a single endosymbiont strain, a single clone and a single wasp species tested under a particular assay, and where the endosymbiont is regarded as symbiotic for its host. Hafer-Hahmann & Vorburger [15] suggest multiple different independent evolutionary interactions might occur within the context of two *Hamiltonella* strains and three wasp species in *A. fabae*. One common wasp, *L. fabarum*, had the least impact on mummification and reproduction when exposed to *Hamiltonella* infected aphids. On the other hand, *A. colemani* affected mummification and reproduction which suggests that it might go extinct after multi-generation wasp exposure. In our situation for *M. persicae*, *D. rapae* (like *L. fabarum* in *A. fabae*) is the primary parasitoid wasp of this species [27,28]. The success of *D. rapae* at the population level could be influenced by a high frequency of *Regiella* if it is established in populations. However, this will also depend on other factors like fitness costs/benefits associated with the endosymbiont independent of interactions with parasitoids. Unlike *Hamiltonella*, the strain of *Regiella* we have used here may have only minor fitness costs (or even benefits) outside of parasitoid interactions when considered as a native infection or a transfection [24,26].

In addition to the above interactions, many other factors will influence the distribution and persistence of endosymbionts within a population, given the diversity of host effects that can be associated with these bacteria. These include the efficiency of vertical transmission and possibility of plant-based horizontal transmission [57], the impact of temperature and other abiotic factors on the endosymbionts and their host effects [58], the impact of host plants [18] and interactions with pathogens, natural enemies and other components of the aphid microbiota [17,59]. An understanding of how such factors interact to influence natural endosymbiont frequencies remains mostly incomplete even for relatively well-studied systems [23].

Understanding endosymbiont–host–parasitoid interactions will be important when evaluating the value of deliberate releases of transinfected or native endosymbionts for pest control [4]. While potential negative effects of novel endosymbionts on mummification rates might be of concern, outcomes need to be considered at the population level where there may be less concern depending on how parasitoids are used in biocontrol. It is nevertheless worth exploring these in detail within the context of resident wasps, host clones and other costs/benefits associated with a particular endosymbiont.

## Supplementary Material



## Data Availability

All data and scripts associated with this manuscript have been archived in a FigShare repository [60]. Modelling and statistical analysis can also be found in the supplementary material.
