# Supplementary material for: Endosymbiotic Regiella insecticola reduce mummification in native and transinfected aphid hosts but do not prevent reproductive damage post parasitism
Source: R Soc Open Sci. 2026 Oct 28;13(10):261936. doi: 10.1098/rsos.261936 (PMC13714403; doi:10.1098/rsos.261936)
Supplement: Supplementary file 1 [file rsos.261936.zip › rsos.261936/rsos261936_si_002.docx]

Figure S1. Scale of confidence in infection of parasitoid DNA and aphid reproduction post exposure to wasps. Each sample screened twice via qPCR with each scale interval representing the level of confidence in parasitoid detection (‘−2’ representing double negative to ‘2’ representing double positive). Each point represents an aphid.

Figure S2. Aphid fitness following exposure to the parasitoid *D. rapae* in relation to their observed stinging status group, *Regiella* infection status, and the presence or absence of parasitoid DNA. Each point represents an individual aphid.
